# Adults with sensorimotor disorders: enhanced physiological and psychological development following specific sensorimotor training

**DOI:** 10.3389/fpsyg.2015.00480

**Published:** 2015-04-22

**Authors:** Mats Niklasson, Peder Rasmussen, Irene Niklasson, Torsten Norlander

**Affiliations:** ^1^Center for Research and Development, Evidens University College, GöteborgSweden; ^2^Vestibularis, Vestibularis Clinic, KalmarSweden; ^3^Department of Neuroscience and Physiology, Institute of Child and Adolescent Psychiatry, Sahlgrenska University Hospital, GöteborgSweden; ^4^Department of Clinical Neuroscience, Karolinska Institutet, SolnaSweden

**Keywords:** DCD, primary reflexes, sensorimotor disorders, sensorimotor therapy (SMT), vestibular stimulation

## Abstract

The aim of the study was to investigate, for the first time, if it is possible to integrate primary reflexes in adults with sensorimotor disorders through sensorimotor therapy (SMT). Participants consisted of 14 adults, one man and 13 women, with an average age of 35 years who completed a SMT program over 3 years. They were compared with a reference group of 100 youngsters spanning from 11 to 17 years. Procedures were the same for both youngsters and adults including regular visits to a therapist and training ~15 min each day at home throughout therapy. Assessments of sensorimotor abilities were made before and after the therapy. Results showed significant improvements on all measurements with regard to treatment for both age groups and the main picture indicated small differences between age groups. After therapy adults were better on balance and orientation tests while the youngsters performed better on sports related gross motor movements, processing of speech sounds and had acquired a better relation between visual skills and vestibular function. Conclusions were that motor problems do not disappear with age and that the same diagnostic instruments and treatment methods can be used for both children and adults with sensorimotor difficulties.

## Introduction

Developmental coordination disorder (DCD) is a neurodevelopmental motor disorder characterized by immature and delayed gross and fine motor development without obvious medical or intellectual causes that noticeably disturbs daily activities (American Psychiatric Association, 2013). Globally, it is calculated to affect between 5 and 13% of schoolchildren (Cairney et al., 2006; Hillier, 2007; Vaivre-Douret, 2014) and there is often a higher incidence in boys as opposed to girls in a ratio of between 2:1 and 7:1 (American Psychiatric Association, 2013). Although there is agreement in respect of the meaning of the concept DCD, still other terms are used more or less synonymously (Ahonen et al., 2004; American Psychiatric Association, 2013) not least because motor difficulties appear in association with several other disorders. According to Gillberg (2010), this co-morbidity within both child psychiatry and developmental medicine is usually the rule rather than the exception. Consequently, DCD can appear together with ADHD (Kadesjö and Gillberg, 1998; Pitcher et al., 2003; Watemberg et al., 2007), as well as with speech difficulties, reading and writing difficulties (Visser, 2003) and with emotional problems (De Raeymaecker, 2006; Cairney et al., 2010). That motor problems do not disappear with age has been shown in a number of studies (e.g., Fox and Lent, 1996; Christiansen, 2000; Rasmussen and Gillberg, 2000; Sigmundsson, 2005) although McPhillips et al. (2000) showed that the reading abilities of participants were positively affected when participants integrated the asymmetrical tonic neck reflex (ATNR) through carrying out stereotypical infant movements. Furthermore, Norlander et al. (2005) showed that a short but regularly performed training program applied to primary and secondary school children addressing the kinesthetic-vestibular systems reduced the noise levels in classrooms and increased the teachers’ rating of the children’s concentration capacity.

The existing definition of DCD (American Psychiatric Association, 2013) does not mention any connection to a sensory cause. What is briefly described though are neurodevelopmental immaturities or neurological soft signs (NSS), which contain a broad scope of neurological deviations, for example, mirror movements, choreiform movements and retained primary reflexes (McPhillips et al., 2000). What these have in common is that they are not considered to belong to any well-defined syndrome, they are difficult to interpret (Touwen, 1993; Polatajko, 1999; Ayd, 2000) and their diagnostic and clinical value is still unclear and therefore further studies are needed (American Psychiatric Association, 2013). At the Vestibularis Clinic, a Swedish center for sensorimotor therapy (SMT), a method for integration of retained primary reflexes (Field and Blythe, 1989) was practiced in order to help children develop their motor abilities. It was, however, observed that motor training became even more efficient when vestibular function was implemented both as a diagnostic tool (Rasmussen et al., 1983; Rasmussen and Gillberg, 2000) and as a part of therapy (Niklasson, 2013) resulting in the method Retraining for Balance (RB; Niklasson et al., 2009, 2010). RB is, in part, a process orientated therapy (Kirby and Sugden, 2007) because it emphasizes the importance of identifying underlying sensory and motor problems, which prevented the individual from developing his or her complete motor skills. In part, it is also an implicit process (Gentile, 1998; Ahonen et al., 2004) where movements activate and organize proprioception as well as vestibular functions. Kirby and Sugden (2007) questioned whether or not DCD is a single syndrome and stressed the complexity of co-morbidity (Visser, 2003; Lingam et al., 2010). Vig (2010) discussed further the importance of labeling and classifying children with disabilities based upon empirical data. Empirical data (Niklasson et al., 2009, 2010) has shown that within the framework of the RB method, it is possible both to diagnose and then treat sensorimotor problems through SMT. Adding the significance of sensory factors, such as vestibular function, for motor development to the existing DCD criteria should increase the possibility of unambiguity in respect of its definition. A tentative suggestion would be that DCD could be more properly labeled as a sensorimotor disorder (SMD). Unpublished studies at the Vestibularis Clinic regarding SMT also call the criteria for DCD into question on the basis that the therapy is also effective for children younger than 5 years of age and for children and young people with intellectual disabilities (Geuze et al., 2001). The present paper will henceforth use the concept SMD.

The primary reflexes, which are essential for an understanding of the phenomenon SMD, consist of around 70 brainstem-mediated behavioral movement patterns (Schott and Rossor, 2003; Niklasson et al., 2009) traditionally considered to belong to early childhood and expected to be integrated by the higher brain centers, which is typical of normal neurological development. Scherzer (1985), Capute and Accardo (1991) as well as Zafeiriou (2004) distinguished primary reflexes that are found in the newborn and which are normally integrated in early childhood from postural reactions, which appear more or less in parallel. Among the primary reflexes they considered as having a diagnostic value were the Moro reflex, the ATNR, the tonic labyrinth reflex (TLR), the grasp reflex in the hands and feet as well as the suck and search reflexes. Important postural reactions were the Landau reaction as well as the head righting reactions. In total, around 15 reflexes were of diagnostic interest. If the primary reflexes do not integrate and if the postural reactions do not develop, there is a risk that the child acquires sensorimotor as well as learning and social difficulties (Goddard Blythe, 2009).

In a naturalistic study (Niklasson et al., 2009) of 232 children and young people (181 boys and 51 girls) between the ages of 5–17 years (*M* = 9.3, SD = 2.7) with concentration problems and motor difficulties who all had followed and completed a SMT program, Retraining for Balance, a number of primary reflexes and postural reactions were studied. On average, the training time was around 3 years and in order to be able to make a comparison between different ages the participants were divided into three groups according to their ages at the start of training: one younger group with 65 children who were 7 years or younger at the start (*M* = 6.2, SD = 2.0); a middle group with 91 children who were 8–10 years (*M* = 9.0, SD = 0.8); and finally a group with older children who were 11 years and older (*M* = 12.3, SD = 1.7). Measured through a number of sensorimotor tests, the results showed significant effects of the training in all three age groups. Biological maturation was suggested by the fact that the older group performed better in comparison with the two other groups before commencing SMT. Furthermore, after the therapy, both the older and middle group performed somewhat better than the younger group. A follow up study (Niklasson et al., 2010) analyzed the results regarding eight children who had completed therapy according to Retraining for Balance. The analysis showed that the process of SMT could be described according to a Kinesthetic-Vestibular Development Model whereby the therapy’s exercises (introductions) gave rise to temporary psychological and/or physical regressions, which were followed by periods of positive psychological and physical development (transformations). In order to examine if the results could be generalized to a larger group of children, the results of the eight participants were compared with the results of the 224 participants who had also completed the training. The quantitative analysis showed that 95% of the children in the larger group had a “very good” or “good” adjustment to the model. Together, both studies indicated that it is seems to be possible to influence sensorimotor (physical) maturity as well as psychological development with children and young people up to 17 years of age by integrating primary reflexes.

Retained or newly appeared primary reflexes are, according to some researchers, a sign of neurological pathology (e.g., Schott and Rossor, 2003; Zafeiriou, 2004) whilst others are of the opinion that the re-appearance of some primary reflexes can be a part of the natural aging process (e.g., Hobo et al., 2014). Touwen (1984) criticized both theories and was of the view that it would be remarkable if a degenerated adult brain could regain the qualities of a child’s brain. The grasp, palmomental, suck, snout and glabellar reflexes, all primitive reflexes with clinical diagnostic value in, amongst others, stroke patients (Chang, 2001) and in patients with dementia and Parkinson’s disease, are activated through tactile stimulation and have, to a limited extent, also been found in healthy adults (e.g., Brown et al., 1998; Schott and Rossor, 2003) but without any link to impaired cognitive abilities (van Boxtel et al., 2006). Several studies (e.g., Bruijn et al., 2013) have made clear that vestibular-proprioceptive connected primary reflexes such as the ATNR and the symmetrical tonic neck reflex (STNR) can be triggered even within healthy adults and can be considered to be natural components in daily motor activities. Also, Easton (1972) stated that primary reflexes could constitute the foundation of sensorimotor organization and Fukuda (1961) wrote that the vestibular system plays an important role for the way we move ourselves as well as for postural development. Clinical conclusions based on retained primary reflexes in adults are, however, a controversial subject (Schott and Rossor, 2003; Zafeiriou, 2004) because primary reflexes are both difficult to interpret and are traditionally associated with some type of brain injury. However, Hurst Vose (1986) has anecdotally described the sensorimotor training she went through after she had been struck by agoraphobia and which resembles the training of children which has been described by McPhillips et al. (2000), Goddard Blythe (2005), and Niklasson et al. (2009). In recent years, research has shown that the brain has previously unknown possibilities of self-healing and restitution after injury (Stein et al., 1997; Kempermann, 2006; Doidge, 2007) and studies have shown that different types of training affect general sensorimotor capabilities of both the elderly and the brain injured (e.g., Beinert and Taube, 2013; Ortega and Jolkkonen, 2013; Patel et al., 2013; Degen and Schröder, 2014; Meunier et al., 2014).

A summary of the various findings lead to the assumption already made by Teicher (1941) in his studies of Schilder’s test (the ATNR and the TLR in standing position) with more than 200 children, namely that, when children approached puberty their movement patterns became more and more like adults. Examples of this can be seen from the fact that minor neurological signs became less pronounced amongst children of 11 years of age (Peters et al., 1975). Additionally, during adolescence the connections in the brain develop, becoming fewer but faster (Giedd et al., 2008) and the nervous system will become more vulnerable to trauma (Eckes and Radunovich, 2007). Furthermore, a summary of earlier research findings indicated that development was described as moving forward (Touwen, 1984; Connolly, 1986; Brodal, 2004; Goldfield and Wolff, 2004; Klonowski, 2007) which means that a physiological return to earlier developmental stages in order to influence maturity becomes hard to explain. Even if the adult’s sensorimotor system nowadays is considered to be plastic (Canu et al., 2012), it is difficult to find in the literature a convincing support for intentional physiological regressions in adults. Therefore, the conclusion may be drawn that it is considerably more difficult for adults to benefit from SMT compared with young people.

The present study is, as far as known, the first attempt to investigate whether primary reflexes can be found in adults voluntarily expressing a wish to be assessed for symptoms of SMDs, and, if so, is it possible to integrate these reflexes through SMT? In light of the above literature review two hypotheses were formulated concerning individuals with SMDs: (a) there are few or no significant differences in terms of sensorimotor abilities among a group of youngsters who are 11 and older and a group of adults 18 years and older *before* completing the SMT, (b) the adult group will exhibit significantly poorer performance compared with the youngsters *after* completing the SMT.

## Materials and Methods

### Participants

The present study included 14 adults and 100 youngsters all of whom had completed a SMT program according to the criteria of the method Retraining for Balance (see Procedure) at the Vestibularis Clinic. Prior to therapy, all participants were diagnosed at the clinic as having SMDs in accordance with the Retraining for Balance-Physiological Test (RB-P) and the Retraining for Balance-Orientation and Balance Test (RB-O; see Instruments). The adult group consisted of one man and 13 women with an average age of 35.21 years (SD = 10.73). The main reasons for therapy when indicated by the adults themselves were sensorimotor problems, often in combination with clumsiness, attention difficulties, reading problems, generalized anxiety, and sensitivity for stress. A reference group of youngsters comprised 72 boys and 28 girls with an average age of 12.44 years (SD = 1.57). Their parents indicated the primary reasons for carrying out therapy and they were similar as compared to the adults. A Pearson Chi-Square showed no significant effects in regard to age groups (*p* = 0.157) concerning reasons for therapy. All participants came from the southern and middle parts of Sweden. On average, the number of visits to the clinic during the therapy program was 13.00 (SD = 2.66) for the adults and 13.61 (SD = 3.02) for the youngsters. An Independent-Samples *t*-test (5 % level) showed no significant difference between groups (*p* = 0.475) regarding number of visits. However, further testing showed a significant difference between groups in terms of time to complete the therapy [*t*(112) = -2.24, *p* = 0.027] where adults needed a longer period (*M* = 37.14 months, SD = 23.24) as compared to the youngsters (*M* = 29.43 months, SD = 9.71).

All tests were performed by one of two therapists/practitioners. Both were Physical Education teachers and SMT-therapists by training with more than 25 years of experience of sensorimotor assessments and training.

### Design

All participants completed a SMT program according to the method Retraining for Balance (Niklasson et al., 2007). The program contained seven parts; (a) fetal and infant movements, (b) vestibular stimulation, (c) auditory perceptual stimulation, (d) tactile stimulation, (e) gross motor milestones, (f) sports-related gross motor skills, and (g) complementary play exercises. The manual described 48 different exercises. Both youngsters and adults practiced about 15 min each day during which the youngsters were monitored by their parents while the adults either practiced alone or were supervised by a relative. The youngster’s training was reviewed by visits to the Vestibularis Clinic at regular intervals of 8 weeks while the adults, from time to time, needed longer intervals between visits due to work and family commitments. The Retraining for Balance program was previously tested and evaluated in 232 children (181 boys and 51 girls) with ages ranging from 5 to 17 years (Niklasson et al., 2009, 2010). The analyses in the present study were conducted through a two-way mixed design with Treatment (before, after) as a within-subjects factor and with Age Group (youngsters, adults) as between-subjects factor. Dependent variables were tests designed to measure sensorimotor abilities.

### Instruments

#### Retraining for Balance-Physiological Test

The RB-P (Niklasson and Niklasson, 2007a; Niklasson et al., 2009) was compiled on the basis of research and documentation of the motor development of normal and developmentally delayed children (Capute et al., 1981; Fiorentino, 1981; Illingworth, 1987; Field and Blythe, 1989; Capute and Accardo, 1991; Holt, 1991). The battery consisted of 41 different tests and the participants’ performance was rated on each test on a 5-point scale from 0 to 4 (“No deviation from normal performance” – “Inability to complete or execute a specific test”). The tests were assembled into six groups generating subscales on (1) Primary reflexes-vestibular stimulation, (2) Primary reflexes-tactile stimulation, (3) Postural reactions, (4) Gross motor milestones, (5) Eye movements, and (6) Sports-related gross motor skills. An index was computed for each group by multiplying the mean by 10, yielding a scale with anchors of 0: “No deviation from normal performance” and 40: “Significant deviation from normal performance.” The six subscales were then added together to form a total value for the RB-P. The instrument has acceptable psychometric properties (Niklasson et al., 2009) with regard to internal consistency and significant correlations with other sensorimotor instruments.

#### Retraining for Balance-Orientation and Balance Test (RB-OB)

This test (Niklasson and Niklasson, 2007b; Niklasson et al., 2009) consisted of balance and vestibular assessments rated according to “no deviation from normal age-appropriate behavior” or “deviation from normal age-appropriate behavior.” The assessments were assembled into three categories, (a) standing balance, (b) vestibular function and (c) body-space perception. A mean was computed for the results in each category and then the categories were added together. A previous study (Niklasson et al., 2009) indicated acceptable psychometric properties for RB-OB.

#### Retraining for Balance – Audiometric Test

This audiometric test, based on a technique developed by Johansen (1993), used the clinical diagnostic audiometer DA 74 (Danaplex, Copenhagen, Denmark). Niklasson et al. (2009) focused on the auditory preference in binaural pure tone audiometry and therefore constructed a scale (RB-A) in order to measure whether the particular participant had a right or left ear preference or whether preference was lacking. The scale spanned 0–200, on which values below 100 indicated left-ear dominance, and values above 100 indicated right-ear dominance. Right ear dominance is supposed to facilitate a more rapid processing of speech sounds (Sininger and Cone-Wesson, 2004). The test’s rationale, namely the importance of right ear-dominance, had been validated by Tallal et al. (1993) and by Okamoto et al. (2007).

#### Reasons for Training (RFT)

A questionnaire (Bergström et al., 1999; Niklasson et al., 2009) was administered after therapy in order to assess the satisfaction of parents and adults. At the start of therapy, on five lines, parents indicated their child’s problems in order of severity and why they thought sensorimotor training was needed. The adults indicated their own problems. Both parents and adults listed as many problems as appropriate. At the end of the therapy the parents and the adults rated how much they thought each problem had changed on a 4-point scale with anchors of 0: No positive change, 1: Little positive change, 2: Quite some positive change, 3: Great positive change. The 4-point scale has been validated (Niklasson et al., 2009) through comparisons with Parent Symptom Questionnaire (Conners, 1973).

#### Keystone Visual Skills Test (KVS)

This is a visual skills test (Burman, 1977) related to vestibular function which has 14 subtests assigned to 15 test cards measuring simultaneous perception, eye coordination, stereovision, as well as the effective acuity during resting accommodation at different distances. The test cards were shown to participants who stated what they saw and responses yielded a maximum of 66 points. The test’s rationale concerning relations between vision and vestibular function had previously been validated (Wenzel, 1978; Braswell and Rine, 2006).

#### The Kinesthetic-Vestibular Development Model (KVDM)

In a previous study, the records of eight children (Niklasson et al., 2010), who had completed therapy according to Retraining for Balance, were examined with the help of qualitative methods. The results indicated that the SMT could be described as a development curve, the Kinesthetic-Vestibular Developmental Model (KVDM), where the exercises push the process forward and create recurrent regressions (negative phases of development), in which three more distinct regressive phases could be discerned. The regressive phases were followed by positive phases of psychological and physiological development where setbacks were transformed into improvements. This is in line with experiences from psychotherapy where such periods of setbacks and progressions are normal (Levin and Gunther, 2003). In order to examine whether the results could be generalized to a larger group of children, the records of the eight participants were compared to the records of 224 children whom had also completed therapy according to Retraining for Balance. Analyses showed that 63% of the children exhibited a “very good adjustment” to the model and 32% of the children showed a “good adjustment” while only 5% were judged to show a “doubtful or poor adjustment” to the model. In the present study, the adjustment to KVDM was rated on a 3-point scale (a) “doubtful or poor adjustment” (only one regressive phase and a few phases of transformation), (b) “good adjustment” (two regressive phases but the main tendency of the model had to be present), (c) “very good adjustment” (three regressive phases and four phases of transformation). Further, the norm group has now been extended to 398 children (aged 4–17 years) treated at the Vestibularis Clinic. The development of all these children has since been rated according to its degree of adjustment to KVDM.

### Procedure

The Vestibularis Clinic has for 25 years been conducting SMT for children with motor problems and attention disorders. Typically, parents had heard about the therapy from other parents, preschool or school administrators or from the school health care provision. Since the method (Niklasson et al., 2009) has not been tested in adults and because it was considered doubtful whether this type of therapy would be of benefit for them, adults have not been admitted to therapy. However, in order to examine whether adults can also benefit from SMT, it was decided to accept a smaller group of them as clients at the clinic. This was done by simply accepting interested adults, who on their own initiative had contacted the clinic and stated a wish to be assessed for SMDs. They had typically heard about the therapy program from other people. The criteria for admittance to the therapy were that participants, through a testing procedure, were diagnosed as having SMDs. When 14 adults had completed the program, it was considered appropriate to conduct a statistical study of the data collected.

In order to have a reference group, previously collected data was used from youngsters in the age group 11–17 years (*N* = 100) who had already been treated at the clinic. In the present study, procedures were the same for both youngsters and adults except for the fact that youngsters were brought to therapy by their parents. An introductory telephone call was always made in order for the therapists to both evaluate the applicant’s medical records and to provide information about the therapy process, including the costs. The fee for the whole program (16 sessions over ca. 3 years) was approximately USD 3.745. How to finance therapy was entirely up to the applicant but mainly fees were paid totally or in part, (a) privately, (b) by the Community Health Service, (c) by the children’s school or by the adult’s employer, or (d) through a foundation. It was also decided whether it was suitable to administer a sensorimotor test (the Physiological Test and the Orientation and Balance Test). An important principle was that neither the youngster’s parents nor the adults admitted to therapy had to sign up for the entire program as participation was voluntary and a decision as to whether to continue with the therapy was made at each visit. Prior to the first visit, the questionnaire Reasons for Training and a mutual agreement was sent to the youngster’s parents or to the adults to be admitted to the therapy.

Three participants in the adult group were on medication in order to increase concentration capacity as compared to none in the youngster group. The medication was in all cases monitored by specialists not connected with the Vestibularis Clinic. At the first visit, the sensorimotor tests were administered by one of the two therapists involved. Participants completed the Physiological Test and the Orientation and Balance Test, which required about 1.5 h. Scores were recorded and participants were informed of the results. A decision on further training was made and instructions for the home training were given. All participants began with the same exercises but as therapy progressed the training became more individually tailored. At the second visit the first Audiometric Test was carried out and at the third visit the first KVS was carried out.

After admittance to therapy, the youngsters were re-tested for sensory and motor performance every 8 weeks during visits of 1.5 h to the clinic. However, due to work and family commitments adults were sometimes re-tested less regularly. Training was performed by the youngsters at home, monitored by their parents, while the adults either trained on their own or together with a relative or a spouse. All participants trained approximately 15 min each day at home throughout therapy. At the last visit, the therapy was evaluated and adults and the youngster’s parents scored the Reasons for Training Questionnaire.

### Ethical Considerations

Before the study was conducted the study procedure was reviewed and approved by the ethical research committee at Evidens University College. The study followed the ethical standards of the World Medical Association’s Declaration of Helsinki concerning Ethical Principles of Medical Research Involving Human Subjects. The clients were informed that written reports would be designed so that their anonymity would be maintained. In addition, each client was informed of his or her right to withdraw from the therapy at any time without having to give a reason. A contract including written consent was drawn up and signed by the client (or a parent) and the therapist.

## Results

### Reasons for Training

Following completion of the program the youngsters’ parents were asked to rate on a 4-point scale the extent of positive changes regarding the problems that constituted their main reason for participation. In the same way the adult participants rated their opinion concerning the effectiveness of treatment. Of all participants, 35 (34.0%) indicated “great positive change,” 56 (54.4%) “quite some positive change,” 9 (8.7%) “little positive change,” and three participants (2.9%) “no positive change.” 11 of the participants did not complete the questionnaire. A Mann–Whitney *U*-test (5% level) showed no significant difference between age groups (*U* = 370, *p* = 0.489).

### Retraining for Balance – Physiological Test

A two-way mixed Pillais’ MANOVA was conducted with Treatment (before, after) as a within-subjects factor and with Age Group (youngsters, adults) as between-subjects factor. The dependent variables were the subscales of the Physiological Test (i.e., Primary reflexes-vestibular stimulation, Primary reflexes-tactile stimulation, Postural reactions, Gross motor milestones, Eye movements, Sports related motor skills) and the RB-P total score. The analyses yielded significant effects for Treatment (*p* < 0.001, η^2^ = 0.78, *power* > 0.99), Age Group (*p* = 0.001, η^2^ = 0.19, *power* = 0.97), and for Treatment × Age Group interaction (*p* = 0.001, η^2^ = 0.18, *power* = 0.97). The results of the univariate *F*-tests with regard to Treatment, Age Group, and Treatment × Age Group are shown below. For means and SDs, see **Table 1**.

**Table 1:** Means (*M*) and standard deviations (SD) for the subscales of the Physiological Test (RB-P), i.e., Primary reflexes-vestibular stimulation (V), Primary reflexes-tactile stimulation (T), Postural responses (P), Gross motor milestones (G), Eye movements (E), Sports related gross motor (S), and the total score for the Physiological Test (Tot) before and after Treatment with the Retraining for Balance program (before treatment = 1, after treatment = 2) with regard to Age group (Youngsters, Adults).

	Youngsters		Adults	
	*M*	SD	*M*	SD
V1	11.77	5.28	12.07	5.81
V2	0.87	1.28	1.38	2.18
T1	3.20	4.07	2.21	3.64
T2	0.66	1.59	0.17	0.34
P1	7.04	4.79	12.06	5.54
P2	0.82	1.66	1.98	2.67
G1	14.01	6.63	17.32	6.39
G2	0.38	1.13	1.01	2.17
E1	13.69	8.69	10.24	7.59
E2	0.97	2.16	2.49	4.36
S1	10.39	8.84	6.90	11.74
S2	0.79	1.92	2.48	4.90
Tot1	60.10	25.53	60.80	28.35
Tot2	4.49	4.96	9.51	12.63

*There were significant improvements on all variables with regard to Treatment for both age groups. In addition, there were significant effects for Age group indicating that the youngsters performed better on Postural responses (P) before and after treatment and on Eye movements (E) before and after treatment. After treatment the youngsters also performed better on Sports related gross motor (S) as compared to adults. Lower values on dependent variables indicate lower levels of sensorimotor disorders.*

#### Treatment

Univariate *F*-tests yielded significant effects for Primary reflexes-vestibular stimulation [*F*(1,112) = 215.91, *p* < 0.001], Primary reflexes-tactile stimulation [*F*(1,112) = 15.25, *p* < 0.001], Postural responses [*F*(1,112) = 154.28, *p* < 0.001], Gross motor milestones [*F*(1,112) = 259.41, *p* < 0.001], Eye movements [*F*(1,112) = 77.19, *p* < 0.001], Sports related gross motor skills [*F*(1,112) = 31.97, *p* < 0.001], and for the RB-P total score [*F*(1,112) = 252.41, *p* < 0.001]. Descriptive analyses showed that in all cases that the participants’ physiological performance improved during the treatment period.

#### Age Group

Univariate *F*-tests yielded significant effects for Postural reactions [*F*(1,112) = 14.13, *p* < 0.001] and Gross motor milestones [*F*(1,112) = 3.98, *p* = 0.048]. There were no other significant differences between age groups in regard of subscales (*ps* > 0.2) or for the total RB-P (*p* = 0.496). Descriptive statistics showed that the youngsters performed better on Postural reactions and Gross motor milestones compared to adults.

#### Interaction Treatment × Age Group

Univariate *F*-tests yielded significant interaction effects for Postural responses [*F*(1,112) = 7.94, *p* < 0.001], Eye movements [*F*(1,112) = 7.93, *p* < 0.001], Sports related gross motor skills [*F*(1,112) = 6.17, *p* = 0.002]. The interaction analyses (Paired-Samples *t*-test, 5% level) showed that both age groups had perceived significant improvement during the treatment period. Further analyses (Independent-Samples *t*-test, 5% level) showed that the two age groups performed at the same level before treatment, but after the treatment the youngsters performed better than the adults in regard to Postural responses, Eye movements, and Sports related gross motor skills.

### Retraining for Balance – Orientation and Balance Test

A two-way mixed ANOVA was conducted with Treatment (before, after) as a within-subjects factor and Age Group (youngsters, adults) as between-subjects factor. The dependent variable was the RB-OB. The analyses yielded significant effects for Treatment [*F*(1,93) = 131.88, *p* < 0.001, η^2^ = 0.59, *power* > 0.99] and for Treatment × Age Group [*F*(1,93) = 5.20, *p* = 0.025, η^2^ = 0.05, *power* = 0.62] but not for Age Group (*p* = 0.733). The interaction analyses (Paired-Samples *t*-test, 5% level) showed that both age groups had perceived significant improvement during the treatment period. Further analyses (Independent-Samples *t*-test, 5% level) showed that the two age groups performed at the same level before treatment, but after the treatment the adults performed better than the youngster in regard to the RB-OB test. For means and standard deviations, see **Table 2**.

**Table 2:** Means (*M*) and standard deviations (SD) for the Orientation and Balance Test (RB-O), the Audiometric Test (RB-A), and the Keystone Visual Skills test (KVS) before and after Treatment with the Retraining for Balance program (before treatment = 1, after treatment = 2) with regard to Age group (Youngsters, Adults).

	Youngsters		Adults	
	*M*	SD	*M*	SD
RB-O1	1.76	0.66	1.95	0.61
RB-O2	0.40	0.57	0.11	0.33
RB-A1	104.32	35.95	74.50	52.02
RB-A2	129.10	32.60	112.23	59.12
KVS1	48.56	8.55	49.71	10.30
KVS2	60.40	5.04	55.08	9.80

*There was significant improvement on all variables with regard to Treatment for both age groups but adults performed better on the RB-O after treatment, while the youngsters performed better than the adults before and after treatment on the RB-A. Regarding the KSV the youngsters performed better after the treatment as compared to the adults. Lower values on the RB-O indicate lower levels of sensorimotor disorders while higher values on the RB-A indicate better processing of speech sounds and the higher values on the KVS indicate a better relation between visual skills and vestibular function.*

### Retraining for Balance – Audiometric test

Before treatment 60 of the youngsters (*valid percent* = 64) had right dominant hearing and after treatment 81 did (*valid percent* = 89). For the adults corresponding values were 6 (*valid percent* = 43) right dominant hearing before treatment and 10 (*valid percent* = 77) after. In order to examine the improvements based on an interval scale, a two-way mixed ANOVA was conducted with Treatment (before, after) as a within-subjects factor and Age Group (youngsters, adults) as between-subjects factor. The RB-A was the dependent variable. The analyses yielded significant effects for Treatment [*F*(1,101) = 20.07, *p* < 0.001, η^2^ = 0.17, *power* = 0.99] and for Age Group [*F*(1,101) = 4.92, *p* = 0.029, η^2^ = 0.05, *power* = 0.59] but not for Treatment × Age Group interaction (*p* = 0.608). Descriptive analyzes showed that both age groups had perceived significant improvement during the treatment period but the youngsters performed better than the adults before and after treatment with regard to right ear dominance based on the RB-A test. For means and SDs, see **Table 2**.

### Keystone Visual Skills Test

In order to examine the improvements based on visual skills, a two-way mixed ANOVA was conducted with Treatment (before, after) as a within-subjects factor and Age group (youngsters, adults) as between-subjects factor and with the KVS as the dependent variable. The analyses yielded significant effects for Treatment [*F*(1,106) = 87.43, *p* < 0.001, η^2^ = 0.45, *power* = 0.99] and for Treatment × Age Group interaction [*F*(1,106) = 4.33, *p* = 0.040, η^2^ = 0.04, *power* = 0.54] but not for Age Group (*p* = 0.12). The interaction analyses (Paired-Samples *t*-test, 5% level) showed that both age groups had perceived significant improvement during the treatment period. Further analyses (Independent-Samples *t*-test, 5% level) showed that the two age groups performed at the same level before treatment, but after the treatment the youngsters performed better than adults in regard to visual skills. For means and SDs, see **Table 2**.

### The Kinesthetic-Vestibular Developmental Model

Information on the degree of alignment to KVDM were rated on a 3-point scale: “doubtful or poor adjustment” (youngsters = 7.0%; adults = 7.7%), “good adjustment” (youngsters = 39%; adults = 46.2%), and “very good adjustment” (youngsters = 54.0%; adults = 46.2%). A Mann–Whitney *U*-test (5% level) with Age Group as independent variable and the ordinal scale (1–3) as dependent variable showed no significant difference between groups (*U* = 601, *p* = 0.616). Finally the two age groups in the present study were compared with a norm group of 398 treated children (ages 4–17) who all had been rated at the Vestibularis Clinic concerning degree of alignment to KVDM (*M* = 2.57, SD = 0.58). There were no significant differences (One-Sample *t*-test, 5% level) concerning degree of alignment between the norm group and the age groups in the present study (youngsters: *M* = 2.47, SD = 0.63, *p* = 0.114; adults: *M* = 2.38, SD = 0.65, *p* = 0.324).

### Handedness of Participants

Of all participants 12.3% had lefthandedness (*n* = 14; males = 12.3%, females = 12.2 %). Of the adults 21.4% had lefthandedness (*n* = 3; one male, two females) and in the youngster group 11% had lefthandedness (*n* = 11; boys = 11.1%, girls = 10.7%).

In order to investigate whether or not there were any significant differences in regard to handedness a split-plot Pillais’ MANOVA was performed with dependent variables of RB-P (total), RB-O, RB-A, and the KVS before and after treatment. Analyses showed no significant results concerning handedness or interaction handedness × treatment effects (*ps* > 0.05).

## Discussion

This study had two hypotheses regarding comparisons between a group of adults and a group of youngsters, both with SMDs, namely (a) there are few or no significant differences in terms of sensorimotor abilities among a group of youngsters who are 11 years and older and a group of adults 18 years and older *before* completing the SMT, and (b) the adult group will exhibit significantly poorer performance compared with the youngsters *after* completing the SMT.

The first hypothesis could, on the whole, be accepted because there were no differences between the adult group and the youngster group in respect of the Physiological Test (RB-P), the Orientation and Balance Test (RB-OB) and the KVS before undergoing SMT. On the other hand, a difference appeared in respect of the Audiometric Test (RB-A) in which the youngsters performed better than the adults before and after therapy with regard to right ear dominance. RB-P and RB-OB are two similar test batteries which overlap each other when it comes to motor and vestibular functions whilst KVS is a complementary test which measures the relationship between visual skills and vestibular function. The results from RB-P and RB-OB are in line with research which has shown that in early puberty the movement patterns of youngsters become more and more like adults (Teicher, 1941; Peters et al., 1975) and that motor difficulties do not disappear with age (e.g., Rasmussen and Gillberg, 2000). Teicher (1941) also showed in his study that there was a great difference between the movement patterns of very young children and older youngsters while sometimes the difference was insignificant between children of similar ages. This difference could also be seen in a later study (Niklasson et al., 2009) where an older group of children (11 years and older), before therapy, performed better on RB-P than both the middle group (8–10 years) and the younger group (7 years and younger). The results of the KVS Tests indicated that visual skills and motor capacities correlated with each other. Previously it has been pointed out that children with sensorimotor problems had visual difficulties (Creavin et al., 2014) and that they ran a greater risk of developing problems with reading and spelling (Lingam et al., 2010).

A previous study (Niklasson et al., 2009) showed no significant differences prior to therapy in respect of RB-A between the three age groups but in the present study the youngsters performed better with regard to right ear dominance than the adults. One reason could be age-related changes (Warren et al., 1978) which make it harder to distinguish with the right ear (Okamoto et al., 2007) whilst other studies (Findlay and Schuchman, 1976; Jerger et al., 1994; Hämäläinen and Takio, 2010) have shown that the preference to use the right ear increases with age. Another reason could be gender-related differences. The present study was comprised of 13 women and only one man. Phillips et al. (2001) showed that women to a higher degree than men use both hemispheres of the brain for passive listening, which could explain why the youngsters initially performed better than the adults. However, a meta-analysis (Sommer et al., 2004) did not show any such significant differences.

The second hypothesis was partly rejected and partly accepted. Although both groups achieved a significantly better result after therapy on the Physiological Test (RB-P), the Orientation and Balance Test (RB-OB), the Audiometric Test (RB-A) as well as on the KVS, the adult group performed significantly worse than the youngsters in tests in respect of postural reactions, eye movements, gross motor milestones and sports- related gross motor skills. The youngsters also achieved better results in respect of the RB-A Test and the KVS Test. On the other hand, having gone through the therapy, the adults performed as well as the youngsters in the RB-P Test as a whole and better than the youngsters in the RB-OB Test. The results are not coherent but still show for the first time that even adults with sensorimotor problems diagnosed as SMDs can integrate primary reflexes through specially adapted therapy. As tentatively suggested by Niklasson (2012), motor development could possibly be an emergent property partly dependent on primary reflex inhibition and vestibular stimulation. If so, primary reflex inhibition and vestibular function (Niklasson et al., 2009) could be the first links in such a developmental chain, followed by emerging postural reactions, gross motor milestones and sports related gross motor skills. The results of the present study showed both that there were no significant differences between the groups when it came to the integrating of primary reflexes and that the adult group performed better when it came to tests linked to vestibular functions.

The results seem to contradict former ideas concerning the possibilities of healthy adults to physiologically regress and transform and further studies are required for an increased understanding of the phenomenon. A tentative approach for further research could be to conceptually connect to physics and chemistry (Prigogine, 1978, 2003; Nicolis, 1993) via dynamic systems theories (Thelen and Smith, 2006) and to the field of epigenetics (Szyf, 2009). Previously, discussing DCD, Kirby and Sugden (2007) argued that a move toward a wider bio-social-educational model from the present narrow medical model would be vital. The reason for the adult group performing significantly worse on some tests after therapy could be due to age-related limitations. If so, the results also indicate the importance of adults, i.e., parents and teachers (Schilder, 1964), understanding the significance of early sensory stimulation of children.

In respect of gender differences, the present study did not permit any analysis but in an earlier study with children and youngsters (Niklasson et al., 2009) there were only a few differences to detect. Tentative speculation suggests that this might be very similar in a larger group of adults. However, an open question is why it was mostly women who voluntarily sought therapy? In the group of youngsters, it was mainly boys and their parents who sought help. A follow up study (Sigurdsson et al., 2002) has previously shown that untreated motor problems are to a higher degree linked to emotional problems in boys as opposed to girls in the teens.

The psychological part of the therapy process (Niklasson et al., 2010) seems to be closely intertwined with the sensorimotor exercises (Niklasson et al., 2009) and can be summarized through the KVDM, which shows how introductions, regressions and transformations in conjunction bring about enhanced physical (sensorimotor) and psychological development. In the present study, there were no significant differences between the group of youngsters and the group of adults in regard to degree of alignment to the KVDM. There were also no significant differences when a norm group was compared with the current groups. A comparison of the way in which children, youngsters and adults react in the way of regressions and transformations shows a similar pattern even if the reactions of the younger often are stronger. Midways in therapy a typical regression among children was an increased wish to be with their mother, often in combination with sadness without obvious reason. Similar behaviors were also described by the adults. One example of this notion was an adult who, in the middle of therapy, saw the back of an old woman reminding her of her deceased mother. She said that the mother had been gone for more than 10 years and that she hadn’t really missed her until now. Grief and a strong longing struck her instantly and she started to weep and had to hurry home. When it came to transformations participants in both groups could after regressions report improved ability to concentrate as well as improved academic achievement. Some of the adults were even able to return to work after long periods of being sick-listed. Periods of regressions and transformations for adults during SMT have not previously been described in the literature, but have been documented among children and youngsters (Niklasson et al., 2010). This might mirror a close and age-independent relationship between certain physiological movements and certain psychological expressions. If these observations are proved right in further studies, SMT might develop into a method for both physical wellbeing and a therapy for psychological development.

The present study had some limitations. One of these is the fact that there was only one man in the adult group. Because participants in the adult group had taken part in the therapy on their own initiative, there was nothing that could be done to increase the number of men participating. Furthermore, the adult group was, in total, small in comparison to the youngster group. These conditions should be remedied in future research. Future research should also comprise normative studies in order to ascertain the occurrence of primary reflexes and vestibular deviations in a normal population.

## Conclusion

The results support both those studies which show that motor problems do not disappear with age and those which show that youngsters’ movement patterns during puberty become more and more like those of adults. Sensorimotor problems in early childhood should therefore be taken seriously (Rasmussen and Gillberg, 2000) and be the target of diagnostic examination and appropriate treatment. The results also show for the first time that the same diagnostic instrument and treatment method can be used for both children and adults with sensorimotor difficulties.

## Conflict of Interest Statement

The authors declare that the research was conducted in the absence of any commercial or financial relationships that could be construed as a potential conflict of interest.
